# Assessment of back proprioceptive sensibility in girls with double curve idiopathic scoliosis

**DOI:** 10.1186/1748-7161-9-S1-O68

**Published:** 2014-12-04

**Authors:** Marianna Białek, Anna Brzęk, Ewelina Białek-Kucharska, Tomasz Kotwicki

**Affiliations:** 1 FITS Center, Jawor, Poland; 2 Institute of Kinesiology, Medical University of Silesia, Katowice, Poland; 3 Department of Pediatric Orthopaedics and Traumatology University of Medical Sciences, Poznan, Poland

**Keywords:** Proprioception, idiopathic scoliosis, FITS method

## Background

Functioning of the proprioceptive system is essential for proper motor control and trajectory planning reflexes, regulation of muscle tension and coordination of muscle activity. Proprioception based biofeedback is used in scoliosis physiotherapy.

## Aim

To assess back skin deep perception in a group of scoliotic girls before and after a 5-day FITS therapy.

## Materials and method

58 girls, aged 10-16 years (mean 13.6 ±1.4) with double curve idiopathic scoliosis were examined. Control group consisting of 40 healthy girls. 12 points were selected at the patient’s back at the scapulae, thorax and waist areas, and a paper sheet with schematic representation of the back was given to each patient. The researcher touched the patient’s back while the patient herself, pointed out the areas where the researcher touched the back. Examination was performed twice: immediately before and after a 5-day FITS therapy.

## Results

In the control group as many as 91.4% girls pointed unerringly to areas located bilaterally near scapulae. Most mistakes occurred when showing the points around waist line on the concave side (34.5% of subjects). In control group most mistakes were made in pointing to places located round the waist line (in 45 to 50% subjects). The size of primary and secondary scoliosis had no influence on the number of errors ( p>0.44). In scoliotic girls undergoing a 5-day FITS therapy, no significant differences were observed (p>0.34).

## Conclusions

• Healthy children scored on average 1.5 point lower than the children with scoliosis.

• No relationship was found between the curvature angle and the accuracy of the task.

• Techniques which stimulate the points with worse proprioception seems worth considering in individual scoliosis therapy.

